# FDG PET/CT and Dosimetric Studies of ^177^Lu-Lilotomab Satetraxetan in a First-in-Human Trial for Relapsed Indolent non-Hodgkin Lymphoma—Are We Hitting the Target?

**DOI:** 10.1007/s11307-022-01731-3

**Published:** 2022-04-29

**Authors:** Ayca Løndalen, Johan Blakkisrud, Mona-Elisabeth Revheim, Jostein Dahle, Arne Kolstad, Caroline Stokke

**Affiliations:** 1 Division of Radiology and Nuclear Medicine, Oslo University Hospital, Oslo, Norway; 2 Institute of Clinical Medicine, Faculty of Medicine, University of Oslo, Oslo, Norway; 3 Department of Physics, University of Oslo, Oslo, Norway; 4 Nordic Nanovector ASA, Oslo, Norway; 5 Department of Oncology, Oslo University Hospital, Radiumhospital, Oslo, Norway

**Keywords:** Indolent non-Hodgkin lymphoma, FDG PET/CT, SPECT/CT, Radioimmunotherapy, Tumor absorbed dose

## Abstract

**Purpose:**

[^177^Lu]Lu-lilotomab satetraxetan, a novel CD37 directed radioimmunotherapy (RIT), has been investigated in a first-in-human phase 1/2a study for relapsed indolent non-Hodgkin lymphoma. In this study, new methods were assessed to calculate the mean absorbed dose to the total tumor volume, with the aim of establishing potential dose–response relationships based on 2-deoxy-2-[18F]fluoro-d-glucose (FDG) positron emission tomography (PET) parameters and clinical response. Our second aim was to study if higher total tumor burden induces reduction in the ^177^Lu-lilotomab satetraxetan accumulation in tumor.

**Procedures:**

Fifteen patients with different pre-dosing (non-radioactive lilotomab) regimens were included and the cohort was divided into low and high non-radioactive lilotomab pre-dosing groups for some of the analyses. ^177^Lu-lilotomab satetraxetan was administered at dosage levels of 10, 15, or 20 MBq/kg. Mean absorbed doses to the total tumor volume (*tTAD*) were calculated from posttreatment single-photon emission tomography (SPECT)/computed tomography (CT) acquisitions. Total values of metabolic tumor volume (*tMTV*), total lesion glycolysis (*tTLG*) and the percent change in these parameters were calculated from FDG PET/CT performed at baseline, and at 3 and 6 months after RIT. Clinical responses were evaluated at 6 months as complete remission (CR), partial remission (PR), stable disease (SD), or progressive disease (PD).

**Results:**

Significant decreases in *tMTV* and *tTLG* were observed at 3 months for patients receiving *tTAD* ≥ 200 cGy compared to patients receiving *tTAD* < 200 cGy (*p* = .03 for both). All non-responders had *tTAD* < 200 cGy. Large variations in *tTAD* were observed in responders. Reduction in ^177^Lu-lilotomab satetraxetan uptake in tumor volume was not observed in patients with higher baseline tumor burden (tTMV).

**Conclusion:**

*tTAD* of ≥ 200 cGy may prove valuable to ensure clinical response, but further studies are needed to confirm this in a larger patient population. Furthermore, this work indicates that higher baseline tumor burden (up to 585 cm^3^) did not induce reduction in radioimmunoconjugate accumulation in tumor.

**Supplementary Information:**

The online version contains supplementary material available at 10.1007/s11307-022-01731-3.

## Introduction

Individualized treatments in modern oncology demand accurate measurement of the pharmaceutical amount reaching the target. Pharmacokinetic (PK) studies are often applied as indirect methods to theoretically determine the distribution both in normal tissue and tumor. Radiolabeled targeted therapies have the advantage of enabling the direct measure of radiopharmaceutical amount accumulating in normal tissue and tumor. Such measurements became more feasible with advances in hybrid imaging technologies.

Targeted therapies like monoclonal antibodies (mAbs) administered as single agents or in combination with other agents have changed the course of non-Hodgkin lymphoma (NHL). Clusters of differentiation (CD) 20 targeting mAb, rituximab, was the first of its kind. Variations in response were reported when rituximab was given as single agent since its introduction [1]. Several studies in early 2000s investigated if this variation may be explained by factors like tumor burden, antigen concentration in tumor, circulating antigens or genetic factors [2, 3]. In recent years, tumor volume measurements have gained increased interest as a parameter to guide individual dose adjustments. Precise measurement of tumor burden before treatment was proposed as part of individualized therapies [4]. Before the introduction of positron emission tomography/computer tomography (PET/CT), tumor burden was solely determined by computer tomography (CT) as the sum of perpendiculars of all lesions, sum of perpendiculars of target lesions or longest diameter of the largest involved node. With the introduction of metabolic tumor volume (MTV) as a 2-deoxy-2-[18F]fluoro-d-glucose (FDG) PET parameter [5], measuring viable tumor volumes has become easier and more precise. MTV can be measured at single lesion level or the whole tumor volume (*tMTV*). Another PET parameter, total lesion glycolysis (TLG), is the product of MTV and the average standardized uptake value (*SUV*_mean_) in the volume of interest. TLG can be calculated at single lesion level or the whole tumor volume (*tTLG*) [6].

Radioimmunotherapy (RIT) works both as targeted radiotherapy and immunotherapy. In addition, it is possible to establish image proof of radioimmunoconjugates successfully targeting the viable tumor mass and to measure the amount of uptake, volume of uptake, and tumor absorbed dose by post-therapy single-photon emission tomography/CT (SPECT/CT). Methods have been proposed to measure the patient mean tumor absorbed dose for ^131^I-tositumomab or Bexxar® (GlaxoSmithKline LLC, Delaware, USA) one of the first RITs approved by the FDA [7–9]. However, to our knowledge, no studies with RIT against indolent NHL have been conducted to investigate the impact of baseline *tMTV*/*tTLG* on radioimmunoconjugate uptake in all tumor tissue and the patient mean total tumor absorbed doses (from here on referred to as total tumor absorbed dose—*tTAD*).

[^177^Lu]Lu-lilotomab satetraxetan or Betalutin® (Nordic Nanovector ASA, Oslo, Norway) has been investigated in the first-in-human phase 1/2a study LYMRIT-37–01 for treatment of relapsed indolent NHL [10]. We have previously investigated absorbed doses to normal tissues, and for selected individual lesions [11, 12]. No absorbed dose–response relationships were then found for single lesions [11]. In the current sub-study of LYMRIT-37–01, we aimed to investigate ^177^Lu-lilotomab satetraxetan radioimmunoconjugate uptake parameters on the whole-body level, and developed method to calculate *tTAD*. The potential therapeutic effect of *tTAD* was then analyzed, based on changes in FDG PET parameters from baseline to 3 and 6 months after treatment (Δ*tMTV*_3months_, Δ*tTLG*_3months_, Δ*tMTV*_6months_, and Δ*tTLG*_6months_) and clinical response after 6 months. Furthermore, we investigated if higher baseline tumor burden (*tMTV*_baseline_) induces reduction in the amount of radiopharmaceutical uptake and tumor absorbed dose.

## Material and Methods

### Patient Characteristics and Treatment

Fifteen patients with relapsed/refractory indolent non-Hodgkin B-cell lymphoma from the multicenter phase 1/2a LYMRIT-37–01 (ClinicalTrials.gov Identifier—NCT01796171) non-randomized trial led by Oslo University Hospital were included in this work. Table 1 shows patient characteristics. Only patients from our center, eligible for dosimetry, were included to assure image standardization. CD37 status of patients were confirmed by immunohistochemistry. Histological subtypes were follicular lymphoma (FL) grade I-IIIA and mantle cell lymphoma (MCL). The LYMRIT 37–01 trial was approved by the regional ethics committee, and all patients had signed an informed consent form.

**Table 1.** Patient characteristics in the entire population. Median values (range) are indicated for continuous variables. Distributions of gender and type of lymphoma are given as number and as percentage

Characteristic		Value
Age (y), median (range)		70 (38–78)
Gender, *n* (%)	Male	13 (87%)
	Female	2 (13%)
Body weight (kg), median (range)		85 (56–111)
Body surface area (m^2^), median (range)		1,99 (1.54–2.35)
Histology, *n* (%)	Follicular lymphoma, grad I	5 (33%)
	Follicular lymphoma, grad II	8 (53%)
	Follicular lymphoma, grad III	1 (7%)
	Mantle cell lymphoma	1 (7%)

**Fig. 1.**
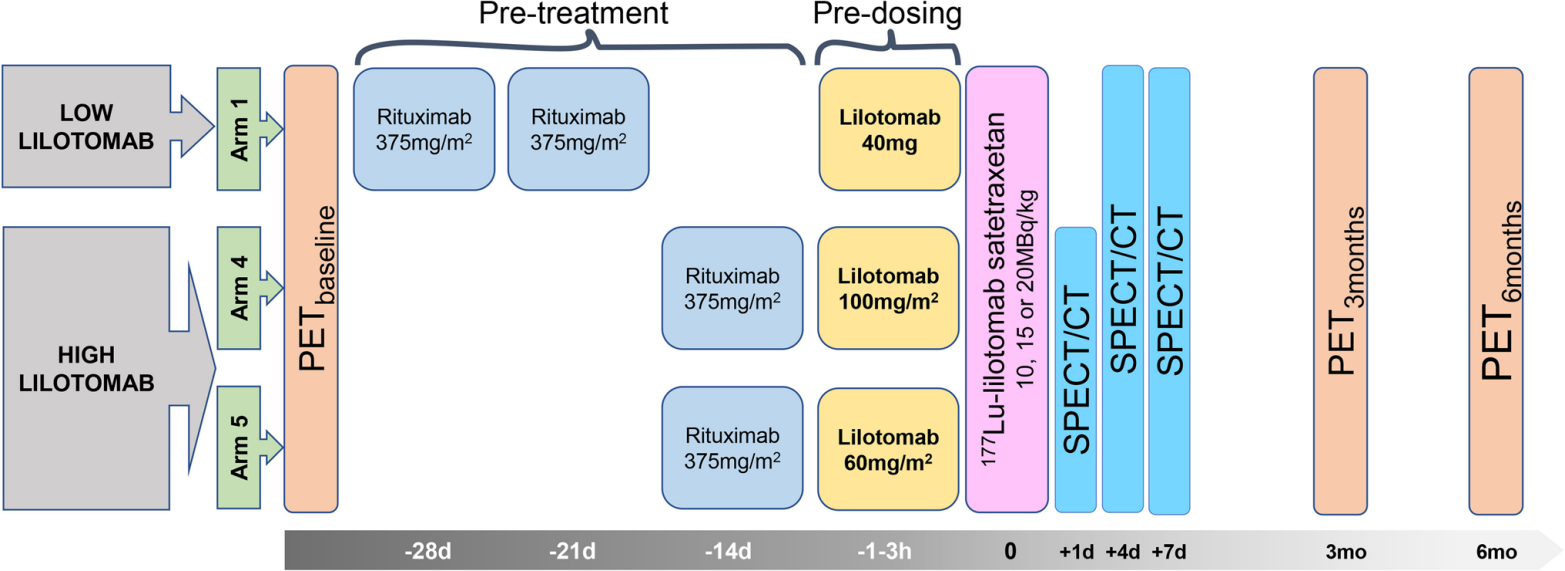
Study design: three different dosage levels, 10, 15, or 20 MBq/kg, were investigated in the LYMRIT-37–01 study. The zero-hour time point on the grey time line indicates administration of ^177^Lu-lilotomab satetraxetan. The current study included arms with three different pre-dosing regimens given 1–3 h before ^177^Lu-lilotomab satetraxetan injection. Based on pre-dosing, patients were here divided into two groups as indicated; low and high lilotomab. Pre-treatment regimens were given 28 and 21 days before or 14 days before the radioimmunoconjugate. FDG PET was performed as baseline investigation and at 3 and 6 months.

Arm 1, 4 and 5 patients at three different dosage levels were included. Arms 2 and 3 without pre-dosing with lilotomab were not included due to the discontinuation of these arms and the limited number of patients in these groups. Patients received a single injection of ^177^Lu-lilotomab satetraxetan; either 10, 15, or 20 MBq/kg body weight. Administered activity: mean 1465 MBq (SD + / − 388) and administered mass: mean 6.4 mg (SD + / − 2.1). All patients were pre-treated with rituximab, and non-radioactive lilotomab was injected as pre-dosing 1–3 h before injection of ^177^Lu-lilotomab satetraxetan (Table 2) (Fig. 1). Patients were also grouped further based on pre-dosing, defining arm 1 with 40 mg lilotomab (standard flat dose to all patients in this arm regardless of body weight and body surface area) as the “low lilotomab” group and arms 4 and 5 receiving 100 mg/m^2^ and 60 mg/m^2^, respectively, as the “high lilotomab” group (Fig. 1).

**Table 2.** Patient treatment. Median value (range) is given for the total injected activity in the entire population. Numbers of patients in each dosage level, stratified by arm, are also given

		Amount	Patients *(n)*
Total injected activity (MBq), median (range)		1434 (746–2189)	15
Injected activity/body weight (MBq/kg)	Arm 1	10	2
		15	2
		20	2
	Arm 4	15	1
		20	7
	Arm 5	20	1

### FDG PET/CT Imaging and Quantification

**Fig. 2.**
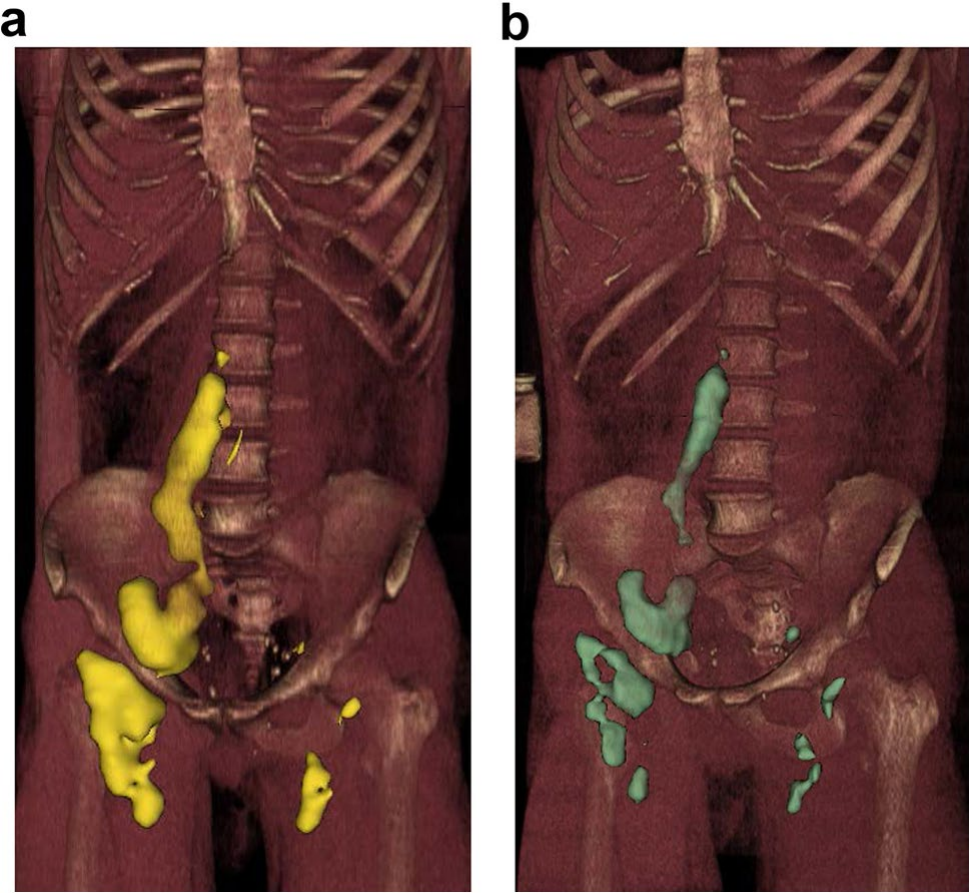
3D renderings of FDG PET/CT and ^177^Lu-lilotomab satetraxetan SPECT/CT images, demonstrating uptake agreement for tumors. **a** PET_baseline_ with all metabolic tumor volumes included. **b** All tumor volumes at day 4 SPECT. Images were reconstructed in 3D for illustration purposes; therefore, physiological uptake was removed from both PET and SPECT.

**Fig. 3.**
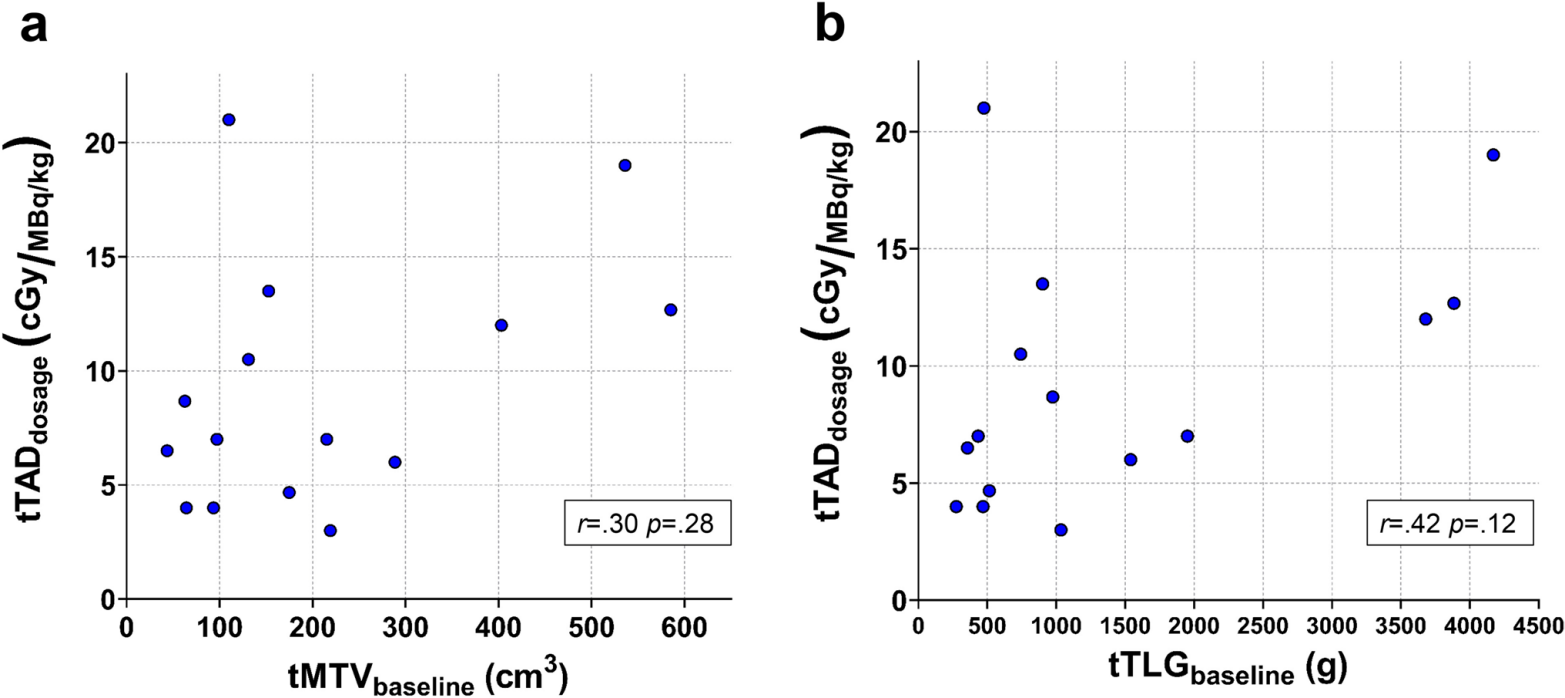
**a** *tTAD*_dosage_ plotted against *tMTV*_baseline_. There was no significant correlation between baseline *tMTV* and *tTAD*_dosage_, implicating that higher *tMTV* did not have a reducing effect on *tTAD*. **b** *tTAD*_dosage_ plotted against *tTLG*. *tTLG* did not correlate with *tTAD*_dosage_. This indicates that absorbed dose cannot be predicted by the FDG uptake at PET_baseline_. The results from the Spearman-rank correlation tests are presented for both analyses.

**Fig. 4.**
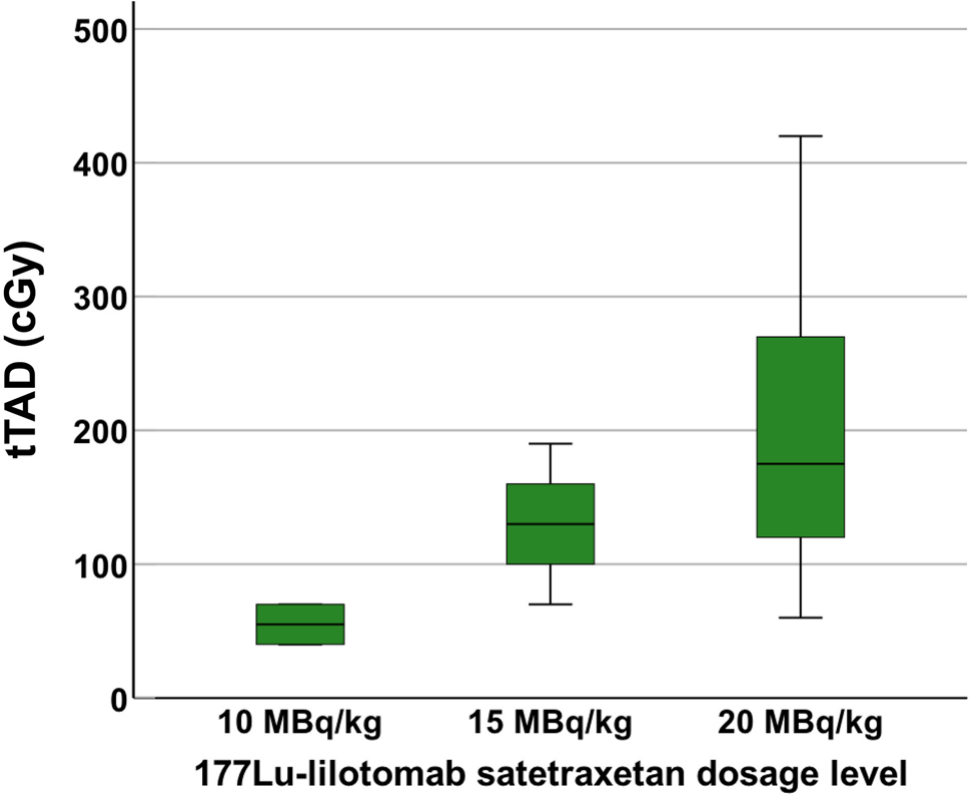
Higher absorbed dose to the total tumor volume, *tTAD*, was observed with increasing ^177^Lu-lilotomab satetraxetan dosage levels. However, the differences in *tTAD* were not significant (*p* = .10). It should be noted that there are 2 patients in the 10 MBq/kg group, which makes this analysis prone to uncertainty.

FDG PET was performed at baseline (PET_baseline_) and repeated 3 months (PET_3months_) and 6 months (PET_6months_) after ^177^Lu-lilotomab satetraxetan treatment. PET/CT images were acquired using a Biograph 16 (Siemens Healthineers) and Discovery MI (GE Healthcare). Acquisitions were performed from vertex to mid-thigh 58–85 min after intravenous administration of 267 to 405 MBq FDG. All PET scans were reconstructed to comply with the EARL standard. *tMTV* and *tTLG* were measured at all three time-points according to EANM procedure guidelines for tumor imaging: version 2 [6]. Syngo.via software solution VB30 (Siemens Healthineers) was used, and a threshold of 41% of *SUV*_max_ applied. Figure 2a illustrates the entire metabolic tumor uptake volume at PET_baseline_ in one of the patients. Changes in these parameters from baseline to PET_3months_ and PET_6months_ were calculated as percent reduction from baseline value, defined as Δ*tMTV*_3months_, Δ*tTLG*_3months_, Δ*tMTV*_6months_, and Δ*tTLG*_6months_. Negative values represent increase in *tMTV* or *tTLG*. All measurements were performed by an experienced nuclear medicine physician. Two patients did not undergo PET_3months_ and PET_6months_ (one of these patients did not undergo contrast enhanced CT (ceCT) either). Data from these patients were used in the analyses regarding the effect of baseline *tMTV*/*tTLG* and effect of dosage levels on *tTAD* (Fig. 3 and Fig. 4. respectively). One patient did not undergo PET_6months_; thus, only PET_3months_ were used in the analyses regarding Δ*tMTV*/Δ*tTLG*.

### SPECT/CT Imaging and Quantification

Patients underwent SPECT/CT at day 4 and day 7 post-injection of ^177^Lu-lilotomab satetraxetan in arm 1 and at day 1, 4, and 7 post-injection in arm 4 and arm 5 (Fig. 1). SPECT/CT scans were acquired with a dual-head Symbia T16 (Siemens Healthineers) scanner. Scanner protocol and reconstruction parameters have been described previously [13]. SPECT/CT data were segmented using the software program PMOD (version 3.6; PMOD Industries) and later post-processed with in-house written python software (version 2.7). Total radioimmunoconjugate tumor volume (*tRTV*) with ^177^Lu-lilotomab satetraxetan uptake was determined on the day 4 and 7 SPECT/CT scans by a semi-automatic approach. An initial manual segmentation was performed by a nuclear medicine specialist to exclude physiological uptake in normal tissue in close proximity to lesions. Then, a thresholding with a 26% cut-off based on the voxel with the highest uptake in the initial segmentation was carried out. This threshold was chosen after a visual optimization that fitted the tumor volumes. The total radioimmunconjugate lesion uptake (*tRLU*) was defined as the total activity inside the *tRTV*. *tRLU* normalized by dosage level was defined as *tRLU*_dosage_ (^*tRLU*^/_dosage level_) (^MBq^/_MBq/kg_). Cumulative activity concentration was calculated by assuming a mono-exponential wash-out of the activity, as previously used for individual tumors [13]. Total tumor-absorbed dose, defined as *tTAD*, was calculated from the time-integrated activity curve and the tumor volume, by assuming a local dose deposition of all electron radiation particles, equating to 0.0853 Gy/(MBqhrs/g) and a tissue density of 1 g/ml [14]. *tTAD* normalized by dosage level was defined as *tTAD*_dosage_ (^*tTAD*^/_dosage level_) (^cGy^_/MBq/kg_).

### Response Assessment

Responses were assessed by FDG PET and ceCT at 3 and 6 months after RIT according to the Cheson criteria [15, 16] defined as complete response (CR), partial response (PR), stable disease (SD) and progressive disease (PD). Bone marrow biopsy was performed to confirm CR if a bone marrow biopsy at baseline was positive. PD was confirmed by CT only.

### Statistics

Spearman-rank correlation tests were performed to investigate relationships between PET and SPECT parameters and between changes in PET parameters and *tTAD*. A significance level of 0.05 was used. The Mann–Whitney *U* test was performed to test differences between groups. The Kruskal–Wallis test was performed to evaluate differences between absorbed doses for the three different ^177^Lu-lilotomab satetraxetan dosage levels. A null hypothesis of equal populations with a rejection level of 0.05 was set for both tests. IBM SPSS version 27 (IBM SPSS Corp) was used for all statistical analysis. Graphpad Prism 8 (GraphPad Software, LLC) and IBM SPSS version 27 (IBM SPSS Corp) were used to create graphs.

## Results

Overall mean (range) imaging-based values were: *tMTV*_baseline_ 212 cm^3^ (44–585 cm^3^), *tTLG*_baseline_ 1427 g (275–4170 g), *tRTV* (day 4) 236 cm^3^ (39–531 cm^3^), *tRLU* (day 4) 18.2 MBq (1.1–56.6 MBq), *tTAD* 170 cGy (40–420 cGy). Mean changes in FDG PET parameters were Δ*tMTV*_3months_ 69% (19–100%), Δ*tTLG*_3months_ 66% (8–100%), Δ*tMTV*_6months_ 50% (− 78 to 100%), and Δ*tTLG*_6months_ 46% (− 134 to 100%) (negative values represent increase). These measures were also stratified by low and high lilotomab groups, as presented in Table 3 and Table 4. Individual values are provided in Supplementary Table 1.

**Table 3.** FDG PET parameters stratified by low and high lilotomab pre-dosing. Mean (range) values are given for each parameter. The *∆* values are calculated from the change relative to baseline, and increases are given as negative values

	*tMTV* baseline (cm^3^)	*tTLG* baseline (g)	∆*tMTV* 3 months (%)	∆*tTLG* 3 months (%)	∆*tMTV* 6 months (%)	∆*tTLG* 6 months (%)
Low lilotomab	138 (63–289)	735 (434–1540)	87 (44–100)	90 (53–100)	79 (7–100)	81 (15–100)
High lilotomab	261 (44–585)	1888 (275–4170)	58 (19–100)	52 (8–100)	30 (− 78 to 100)	21 (− 134 to 100)

**Table 4.** SPECT/CT parameters stratified by low and high lilotomab pre-dosing. Mean (range) values are given for each parameter. *tRTV*, *tRLU*, and *tRLU*_dosage_ in the first three columns are day 4 values

	*tRTV* (cm^3^)	*tRLU* (MBq)	*tRLU*_dosage_ (^MBq^/_MBq/kg_)	Effective half-life for *tRLU* (days)	*tTAD* (cGy)	*tTAD*_dosage_ (^cGy^/_MBq/kg_)
Low lilotomab	141 (39–219)	6.2 (1.1–9.8)	0.4 (0.1–0.7)	3.2 (1.7–4.8)	142 (40–420)	8.6 (4.0–21.0)
High lilotomab	298 (114–531)	26.1 (6.4–56.6)	1.4 (0.3–3.0)	3.2 (2.7–3.8)	189 (60–380)	9.8 (3.0–19.0)

Tumor volumes on PET_baseline_ (*tMTV*_baseline_) and SPECT day 4 and day 7 (*tRTV *- day4 and day7) correlated significantly (both *p* < 0.01) as expected. Supplementary Fig. 1a shows data for *tRTV *- day4. Interestingly, there were also strong correlations between glucose consumption, *tTLG*_baseline_, and radioimmunoconjugate uptake normalized by dosage, *tRLU*_dosage _- day4 and day 7 (both, *p* < 0.01), an indication that ^177^Lu-lilotomab satetraxetan successfully targets FDG avid tumor tissue. Supplementary Fig.1b shows data for *tRLU*_dosage_ day 4. However, radioimmunconjugate activity concentration (expressed as $^{tRLU_{\mathrm{dosage}}}{_{/\mathrm{volume}}}$) and baseline *SUV*_mean_ correlation were not significant (*p* = 0.07), indicating that consumption of glucose and CD37 expression on tumor cells does not correspond (Supplementary Fig. 1d).

We tested if increasing baseline tumor volumes have reducing effect on radioimmunoconjugate uptake, a probable sign of antibody shortage for higher target antigen burden. A significant positive correlation between *tRLU*_dosage_ and *tRTV* indicates that the total tumor uptake of radioimmunconjugate does not decrease, but contrarily increases with larger tumor volumes (*p* < 0.01) (Supplementary Fig. 1c). Another way of testing this was by analyzing the correlation between *tMTV* and *tTAD*_dosage_. This analysis demonstrated that *tTAD*_dosage_ increased slightly with larger *tMTV*_baseline_ (Fig. 3a). Even if the correlation was not significant, it is still indicating that larger tumor volumes probably do not cause shortage of radioimmunoconjugate. A similar trend was observed between glucose consumption (*tTLG*_baseline_) and *tTAD*_dosage_ (Fig. 3b).

Higher total tumor absorbed doses (*tTAD*) were observed with increasing ^177^Lu-lilotomab satetraxetan dosage levels, but the differences were not significant (*p* = 0.10). It should be noted that there are 2 patients in the 10 MBq/kg group which makes this analysis prone to uncertainty (Fig. 4).

*tTAD*_dosage_ was slightly higher in the high lilotomab group (Table 4), but the differences were not significant across low and high lilotomab groups (*p* = 0.61).

**Fig. 5.**
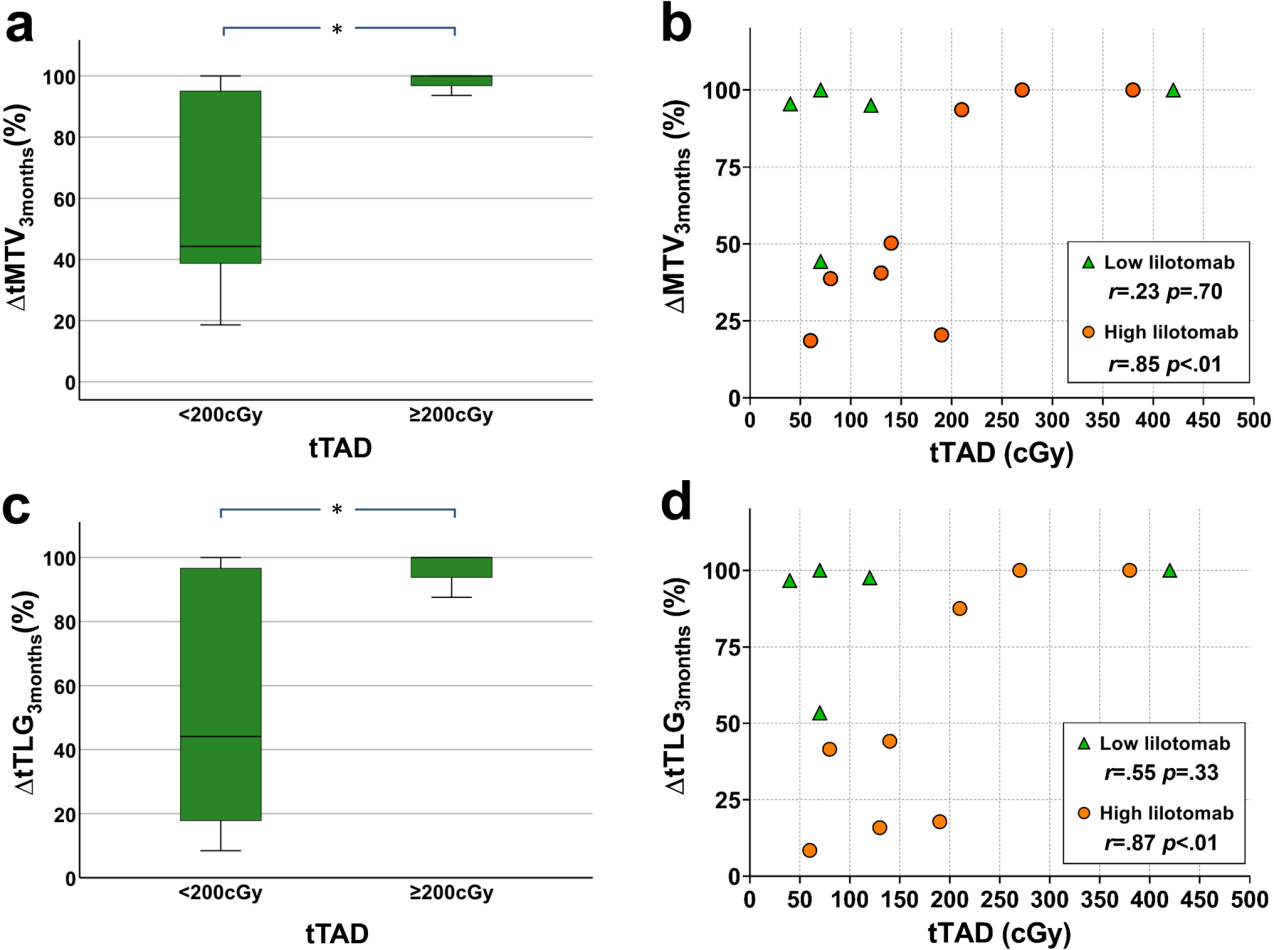
**a** Boxplot demonstrating significantly higher ∆*tMTV*_3months_ for patients with *tTAD* ≥ 200 cGy compared to group with < 200 cGy (*p* = .03). **b** ∆*tMTV*_3months_ plotted against *tTAD* for the high and low lilotomab groups. **c** Boxplot demonstrating significantly higher ∆*tTLG*_3months_, for patients with *tTAD* ≥ 200 cGy compared to group with < 200 cGy (*p* = .03). **d** ∆*tTLG*_3months_ plotted against *tTAD* for the high and low lilotomab groups. **a** and **c** Demonstrate large variations in ∆*tMTV*_3months_ and ∆*tTLG*_3months_ for *tTAD* < 200 cGy, while a more predictable ∆*tMTV*_3months_ and ∆*tTLG*_3months_ was observed for *tTAD* ≥ 200 cGy. Significant differences annotated by asterisks. **b** and **d** Demonstrate increases in ∆*tMTV*_3months_ and ∆*tTLG*_3months_ with increasing *tTAD* in the high lilotomab group indicating significant tumor shrinkage with higher *tTAD*. This could not be demonstrated in the low lilotomab group. It may be that the overall good response in this group masks such a correlation. The results from the Spearman-rank correlation tests are presented in **b** and **d** for each group. Each symbol represents an individual patient.

Reduction in metabolic tumor volumes (Δ*tMTV*_3months_) and glucose consumption (Δ*tTLG*_3months_) after RIT were significant for the *tTAD* ≥ 200 cGy group compared to the group receiving < 200 cGy (*p* = 0.03) (Fig. 5a and c). A similar correlation was shown at PET_6months_ (Δ*tMTV*_6months_ and Δ*tTLG*_6months_) but did not reach significance (*p* = 0.07 for both) (data not shown).

Tumor volume shrinkage and decrease in glucose consumption expressed as Δ*tMTV*_3months_, Δ*tTLG*_3months_, Δ*tMTV*_6months_, and Δ*tTLG*_6months_ were statistically significantly correlated with increasing *tTAD* in the high lilotomab group. Such correlation could not be demonstrated in the low lilotomab group (Fig. 5b and d for Δ*tMTV*_3months_, Δ*tTLG*_3months_, respectively) (data not shown for Δ*tMTV*_6months_, Δ*tTLG*_6months_). However, higher mean Δ*tMTV*_3months_, Δ*tTLG*_3months_, Δ*tMTV*_6months_, and Δ*tTLG*_6months_ were observed in this group, and the lack of a correlation can be explained by the small variations in response (Table 3).

**Fig. 6.**
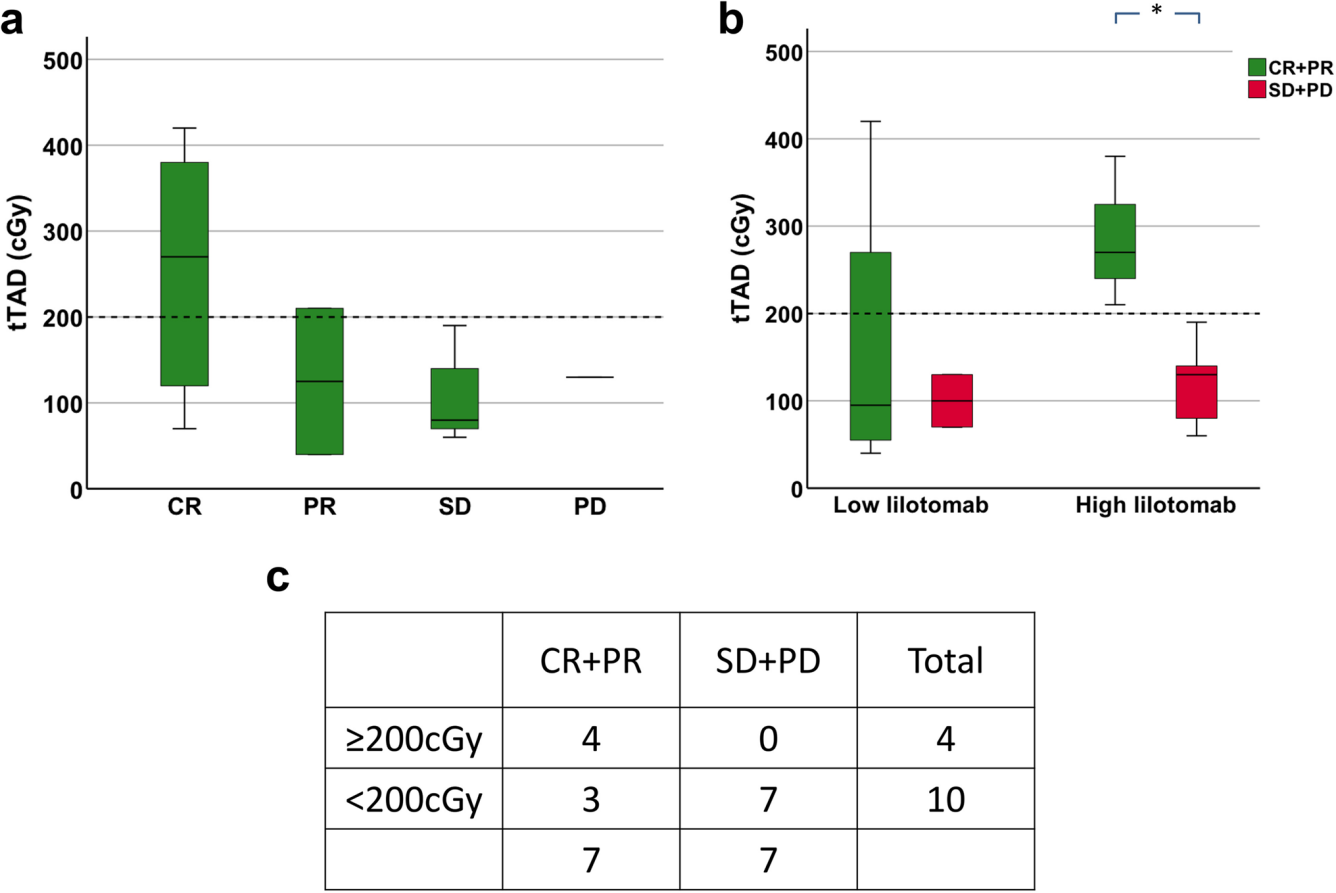
**a** Absorbed dose to the total tumor volume, *tTAD*, in the four clinical response categories. Higher *tTAD* was observed in patients with CR, compared to SD and PD. **b** *tTAD* for response categories grouped as responders (CR + PR; in green) and non-responders (SD + PD; in red), and further stratified by low and high lilotomab. Responders had a significantly higher *tTAD* than non-responders in the high lilotomab group (*p* = 0.04). This could not be demonstrated in the low lilotomab group (*p* = 1.0). The latter had large variations in *tTAD* in responding patients, and only two patients were non-responders in this group, which makes this analysis prone to uncertainty. Significant difference annotated by asterisks. **c** Responders and non-responders stratified by a 200 cGy threshold. All non-responders had *tTAD* < 200 cGy, while all with *tTAD* ≥ 200 cGy were responders. Overall large variations in *tTAD* were observed in responders.

Five patients had CR, two had PR, five had SD, and two had PD (Fig. 6a and Supplementary Table 1). *tTAD* was statistically significantly higher in responders (CR + PR) compared to non-responders (SD + PD) in the high lilotomab group (*p* = 0.04) but not in the low lilotomab group (*p* = 1.0) (Fig. 6b), similar to the results from Δ*tMTV* / Δ*tTLG* analyses. Large variations in *tTAD* were observed in responders in low lilotomab group (range 40–420 cGy) (Fig. 6b) (Supplementary Table 1). Across the entire cohort, independent of amount of pre-dosing, all non-responders had *tTAD* < 200 cGy; however, large variations in *tTAD* were observed in responders; especially in the low lilotomab group (Fig. 6).

## Discussion

In this era of precision medicine and personalized therapy, it is imperative to explore the best way of delivering a treatment with precise dosing tailored for each individual patient. Although time-consuming, tumor and normal tissue dosimetry is a crucial part of targeted radiotherapies, and should be standard both in the clinical setting and in trials. Radioimmunoconjugate uptake determined by post-therapy SPECT-derived metrics is an accurate method of analyzing the amount of radioactivity accumulating in tumor; an option unavailable for non-radioactive mAb treatments. In this sub-study of LYMRIT-37–01, the total amount of ^177^Lu-lilotomab satetraxetan accumulated in tumor (*tRLU*), total tumor uptake volume (*tRTV*), and total tumor absorbed doses (*tTAD*) were calculated from post-therapy SPECT/CT. Our results indicate that ^177^Lu-lilotomab satetraxetan targets FDG avid tumor tissue without a reduction in uptake in larger tumor volumes; hence, no indication of radioimmunoconjugate shortage was found. Furthermore, especially for the high lilotomab group, *tTAD* showed an impact on both Δ*tMTV* and Δ*tTLG*, and on clinical response.

Standard PK methods to theoretically calculate the amount of a radiopharmaceutical reaching the tumor volumes outside blood compartment without molecular imaging-based support is not straightforward. This is mainly because of changes in biodistribution between tumor and normal tissue as shown by Stokke et al. for ^177^Lu-lilotomab satetraxetan [12]. Image-based measurement of the amounts accumulating in the tumor mass is feasible for targeted radiotherapies where it also enables the calculation of tumor absorbed doses. Despite this advantage, tumor dosimetry is still an underutilized method. From such measurements, several interesting findings were derived for ^177^Lu-lilotomab satetraxetan in this work. A strong correlation between *tRLU*_dosage_ and *tRTV* implicates that increasing tumor volumes do not reduce ^177^Lu-lilotomab satetraxetan accumulation in tumor (Supplementary Fig. 1c). This was also demonstrated by larger *tMTV*_baseline_ not resulting in reduced *tTAD*_dosage_ (Fig. 3a). It is therefore fair to assume that the injected amount of radioimmunoconjugate was sufficient for all tumor volumes studied and larger tumor volumes of up to 585 cm^3^ do not result in shortage of ^177^Lu-lilotomab satetraxetan. Recent PK studies have reported that tumor burden influences availability of two different CD20 mAbs, rituximab and obinutuzumab, in NHL patients. It was proposed that the standard dose given may not reach sufficient therapeutic levels of mAbs in cases with high tumor burden [4, 17, 18]. While reduction of *tRLU* or *tTAD* with increasing tumor burden was not demonstrated in our study, a lower mean tumor volume (212 cm^3^) in our population compared to Tout et al. (313 cm^3^) [4] and Ternant et al. (600 cm^3^) [18] might explain why we did not observe such effects. However, Ternant et al. used different methodology to measure *tMTV*; thus, a direct comparison with our study is not possible. Different levels of CD20 and CD37 expressed by cells, and different administration protocols and pharmacological properties of rituximab versus ^177^Lu-lilotomab satetraxetan hinder direct comparisons. By another approach, whole body (WB) absorbed doses for ^131^I-tositumomab were used to demonstrate availability of radioimmunoconjugate. By this method, dosing and pre-dosing regimens and the possibility of fractionation to reach high WB absorbed doses and longer half-life of radioimmunoconjugate were evaluated [19]. Changes in biodistribution after different pre-dosing regimens have previously been demonstrated for ^177^Lu-lilotomab satetraxetan [12]. Thus, the approach using WB absorbed doses is probably not precise enough to reflect the amount reaching the tumor for ^177^Lu-lilotomab satetraxetan.

Application of *tTLG* in treatment planning or changes in *tTLG* to evaluate response during, and after treatment in lymphoma has been proven useful [20, 21]. In our study, lack of correlation between baseline *tTLG* and *tTAD*_dosage_ indicates that absorbed dose cannot be predicted by FDG uptake intensity at baseline FDG PET (Fig. 3b). There was strong correlation between *tTLG* and *tRLU*_dosage_ (Supplementary Fig. 1b), but activity concentration defined by$^{tRLU_{\mathrm{dosage}}}{_{/\mathrm{volume}}}$ and *SUV*_mean_ (calculated across the total tumor tissue) was not significant (Supplementary Fig. 1d). Thus, the *tTLG*_baseline_ vs *tRLU*_dosage_ correlation can possibly be attributed to the fact that these parameters were derived from their respective volumes rather than a similarity between consumption of glucose and CD37 expression on these cells. While this still supports that ^177^Lu-lilotomab satetraxetan successfully targets the viable tumor cells in the volume of interest determined from baseline FDG PET, it also indicates that FDG uptake intensity does not necessarily correlates with CD37 expression in tumor.

We have previously investigated lesion-based tumor-absorbed doses and dose–response relationships, by analyzing 1–5 selected lesions per patient [11]. The criteria for lesion inclusion were then strictly defined for individual dosimetry of each tumor. Significant intra-patient variations were observed and absorbed dose–response relationship at lesion level could not be demonstrated based on changes in FDG PET parameters and Deauville 5-point-scale [11]. In the current study, by measuring *tTAD*, we averaged out intra-patient variations and most importantly avoided possible selection bias. In addition, arms 2 and 3 without pre-dosing with lilotomab were not included to assure a more homogenous group which can be analyzed as one, for some of the analyses. Traditionally, radioimmunotherapy of lymphoma includes pre-dosing with non-radioactive mAbs; therefore, comparisons with earlier studies are assumed to be more accurate by including only patients receiving non-radioactive mAb as pre-dosing before treatment. While it can be argued that mean absorbed dose is not an adequate metric, and that local low-dose areas are relevant for the overall response, this parameter has been demonstrated as a significant predictor for ^131^I-tositumomab treatment [7, 8]. Mean *tTAD* in our study was 170 cGy (median 130 cGy). This is lower than the median value of between 341 and 275 cGy reported with ^131^I-tositumomab (Bexxar®) by Dewaraja et al. [7, 8]. Methodologies applied in these two studies are partly comparable to ours, although the CT-driven approach for tumor delineation, performed for ^131^I-tositumomab, can potentially result in a lower mean tumor absorbed dose (i.e. *tTAD*) compared to our current method which may exclude tumor tissue with very low uptake. Also, post-therapy dosimetry was based on imaging at day 2, 5, and 7–9 for ^131^I-tositumomab and day 4 and 7 in the present study. While imaging data for day 1 were available for arm 4 and 5, this time-point was not included in the dosimetry calculation due to harmonization between arms. While a previous publication showed the mean difference between 2 and 3 time-points to be 5.5% (maximum error 16%) [13], this is a possible limitation in the current work. In addition, Dewaraja et al. took into account the non-radioactive antibody effect which we did not because of limited cell killing effect of lilotomab demonstrated by *in-vitro* cell studies [8, 22].

Based on the proposal by Dewaraja et al. [8], we decided to pursue a 200 cGy *tTAD* threshold by investigating the changes in FDG PET parameters and response status stratified by this threshold in our population. ∆*tMTV*_3months_, ∆*tTLG*_3months_, ∆*tMTV*_6months_, and ∆*tTLG*_6months_ were higher in *tTAD* ≥ 200 cGy group and this difference was significant for ∆*tMTV*_3months_ and ∆*tTLG*_3months_ (Fig. 5a and c), indicating that there is indeed an absorbed dose–response correlation also for ^177^Lu-lilotomab satetraxetan and that the same threshold can be applied. All four patients with *tTAD* ≥ 200 cGy had ∆MTV_3months_ ≥ 90%. Variations in response in the lower *tTAD* (< 200 cGy) group were larger. While the patient with the lowest *tTAD* (37 cGy) had ∆MTV_3months_ = 96% and ∆MTV_6months_ = 89%, a patient with progression (∆MTV_6months_ =  − 77%; negative value represents increase) had *tTAD* = 100 cGy. One of the patients with progressive disease was the only mantle cell lymphoma in our study with *tTAD* = 77 cGy. Even though mantle cell lymphomas have been characterized as radiosensitive [23], like follicular lymphomas, this patient unfortunately did not respond to ^177^Lu-lilotomab satetraxetan treatment. There are few patients in our study and these dissident findings may be random, but it is likely that absorbed doses ≥ 200 cGy gives a more predictable effect, whereas the response to lower absorbed doses (< 200 cGy) may be more dependent on individual radiosensitivity. While the threshold of 200 cGy may seem low, it is also in relative accordance with low dose involved field external beam radiotherapy (2 × 2 Gy) inducing high response rates for indolent lymphomas [24]. Even if direct comparisons with external beam radiotherapy cannot be made due to different beam qualities, dose rates, etc., this is in the same order of magnitude.

When analyzing the effect of pre-dosing on absorbed doses, we observed a slight but not significantly higher *tTAD*_dosage_ and *tTAD* in high lilotomab group. Interestingly, mean Δ*tMTV*_3months_, Δ*tTLG*_3months_, Δ*tMTV*_6months_, and Δ*tTLG*_6months_ were lower in this group despite slightly higher *tTAD* (Table 3 and 4). A clear dose–response relationship was illustrated for this group, with higher *tTAD* inducing statistically significant metabolic tumor volume shrinkage and reduction in lesion glycolysis (Fig. 5b and d for Δ*tMTV*_3months_ and Δ*tTLG*_3months_. Data not shown for 6 months data). On the contrary, the low lilotomab group with slightly lower *tTAD*_dosage_ and *tTAD* had higher mean Δ*tMTV*_3months_, Δ*tTLG*_3months_, ΔMTV_6months_ and Δ*tTLG*_6months_ (Table 3 and 4). Dose–response relationships could not be demonstrated in this group (Fig. 5b and d). This is expected since the overall high response rate could mask a possible dose–response relationship. Why such a difference in response as higher mean ∆*tMTV*_3months_, ∆*tTLG*_3months_, ∆*tMTV*_6months_, and ∆*tTLG*_6months_ was observed in low lilotomab group and whether other factors that may influence the response are still open questions. A possible explanation may be the differences between baseline mean *tMTV* between low and high lilotomab groups (Table 3). However, the differences were not significant in the current population (*p* = 0.27).

The LYMRIT 37–01 PK study demonstrated an increase in blood activity adjusted exposure (area under the curve) with higher lilotomab pre-dosing levels. According to this PK analysis, arm 4 (high lilotomab) demonstrated the highest exposure, the lowest clearance, and the longest biological half-life of ^177^Lu-lilotomab satetraxetan, slightly higher than arm 1 (low lilotomab) [10]. Furthermore, lower bone marrow and spleen absorbed doses in arm 4 [12] in addition to higher blood exposure shown by PK study [10] indicates that more ^177^Lu-lilotomab satetraxetan is available for tumor uptake in this arm. This proposed effect was supported in our study by slightly higher *tTAD*_dosage_ in the high lilotomab group (arm 4 and 5), even though this was not significant. Larger *tTAD*_dosage_ variations were also observed in the high lilotomab group, in line with our previous lesion-based tumor-absorbed dose analysis [11].

Evaluation of clinical response versus *tTAD* also supports the assumption of absorbed dose–response relationships and a 200 cGy threshold. Patients with CR had large variations in *tTAD* (range 69.5–418.3 cGy) (Supplementary Table 1), while all patients with SD or PD had *tTAD* < 200 cGy (Fig. 6a and c). Only two patients had PR; one just above a *tTAD* of 200 cGy and one below. Notably, all patients with *tTAD* ≥ 200 cGy were responders, whereas all non-responders had *tTAD* < 200 cGy (Fig. 6c). Based on this analysis, we propose a threshold of 200 cGy to ensure CR, while for < 200 cGy large variations in response may be expected. Our methodology for *tTAD* can exclude tumor volumes with low uptake. However, the inclusion of low uptake tumor volumes ensures not to overestimate the patients’ mean tumor absorbed doses. This means that our conclusions with respect to the 200 cGy limit are conservative and can be safely employed regardless of methodology. Applying a different approach, resulting in lower *tTAD*s, would not misplace any < 200 cGy patients in the ≥ 200 cGy group (only CR). Thus, the observation that all non-responders had *tTAD* < 200 cGy would also hold true using a different approach. When comparing responders and non-responders in low and high lilotomab groups, a similar pattern as for the PET response evaluation was revealed. *tTAD* was statistically significantly higher in responders (CR + PR) compared to non-responders (SD + PD) in the high lilotomab group (*p* = 0.04). In the low lilotomab group, the response rates were generally higher, and there were only two patients with SD + PD (Fig. 6b). The reason for the difference between the high and low lilotomab groups is not clear, as discussed above, but regardless of pre-dosing, all non-responders had *tTAD* < 200 cGy.

We observed increasing *tTAD* with increasing ^177^Lu-lilotomab satetraxetan dosage levels in this study (Fig. 4), but the differences were not significant (*p* = 0.1). This illustrates that increasing the amount of activity administrated will not necessarily increase the absorbed dose significantly as this value will also depend on patient-specific uptake and kinetics. Δ*tMTV*_3months_, Δ*tTLG*_3months_, Δ*tMTV*_6months_, and Δ*tTLG*_6months_ did not either vary between the 3 dosage levels (*p* = 1, *p* = 1, *p* = 0.8, and *p* = 0.8 respectively), but notably, there was a difference for these parameters according to *tTAD* with threshold 200 cGy, as discussed above. This finding indicates that response does not necessarily directly rely on dosage levels, and that absorbed dose can be further investigated as a solitary predictor.

## Conclusion

In this study, ^177^Lu-lilotomab satetraxetan total tumor absorbed doses were calculated and an absorbed dose–response relationship in indolent NHL patients was revealed in the high lilotomab pre-dosing group. Our results suggest that prediction of response with tumor absorbed doses ≥ 200 cGy is reasonable, while large variations of response should be expected for tumor-absorbed doses < 200 cGy.

Higher baseline tumor burden did not induce reduction of ^177^Lu-lilotomab satetraxetan uptake in tumor, indicating that the amount of radioimmunoconjugate given was sufficient for all tumor volumes studied. However, further studies are needed to establish this in a patient population with a larger range of volumes.

Well-designed dosimetric studies are the most direct method to measure the uptake of radioimmunoconjugates in targeted radiotherapies. This provides valuable information to determine the optimal dosage levels and pre-dosing regimens to attain the highest possible absorbed dose to tumor while maintaining acceptable absorbed doses to normal tissues.

## Supplementary Information

Below is the link to the electronic supplementary material.

Supplementary file1 (DOCX 268 KB)

Supplementary file2 (DOCX 51 KB)
