# Supplementary material for: FDG PET/CT and Dosimetric Studies of 177Lu-Lilotomab Satetraxetan in a First-in-Human Trial for Relapsed Indolent non-Hodgkin Lymphoma—Are We Hitting the Target?
Source: Mol Imaging Biol. 2022 Apr 29;24(5):807–17. doi: 10.1007/s11307-022-01731-3 (PMC9581842; doi:10.1007/s11307-022-01731-3)
Supplement: Supplementary file 1 — Supplementary file1 (DOCX 268 KB) [file 11307_2022_1731_MOESM1_ESM.docx]

**
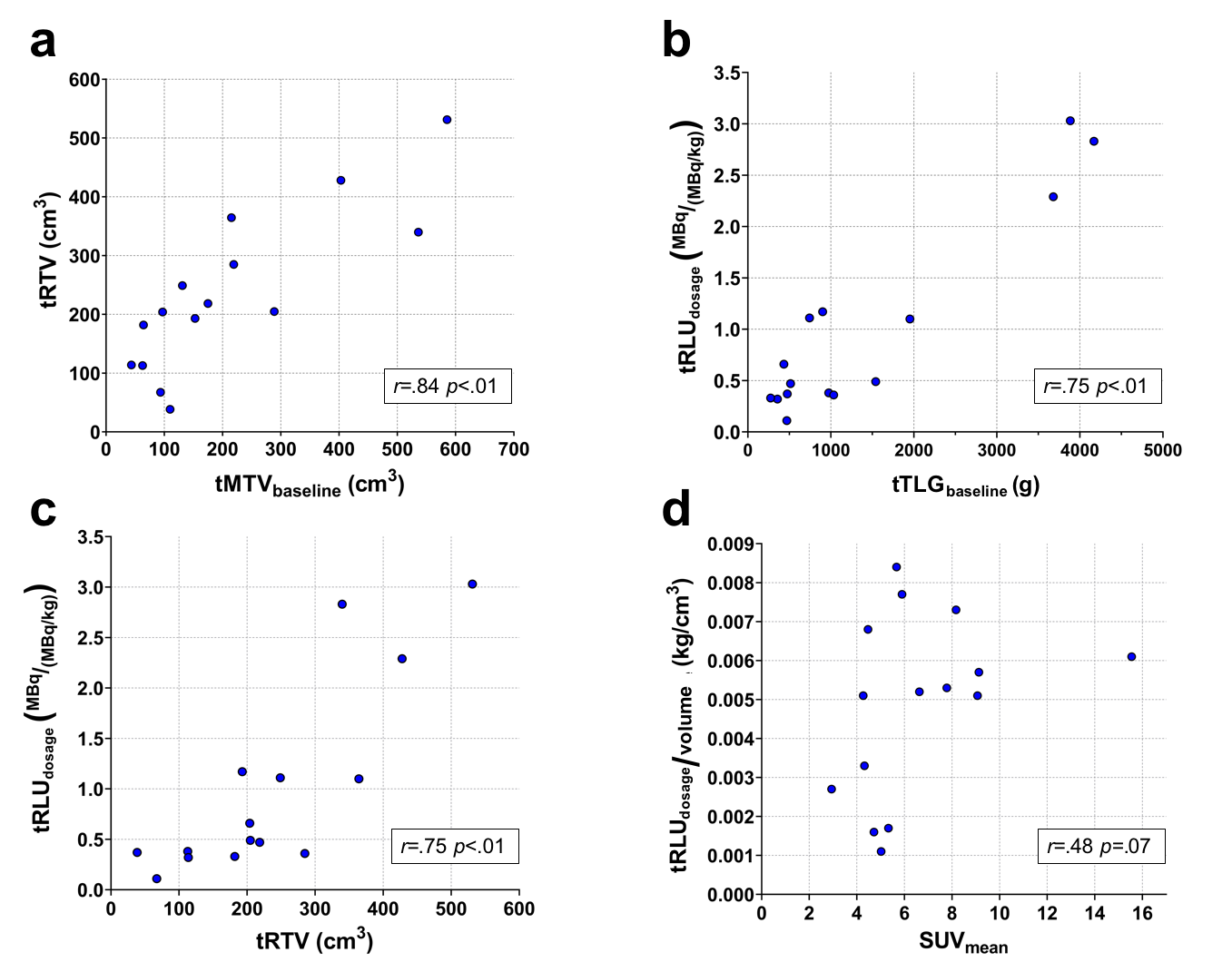
Supplementary Fig. 1.** **(a)** The ^177^Lu-lilotomab satetraxetan uptake volume tRTV plotted against tMTV_baseline_. A statistically significant correlation between the volumes tMTV_baseline_ and tRTV was found as expected **(b)** The ^177^Lu-lilotomab satetraxetan uptake normalized by dosage level, tRLU_dosage_, plotted against tTLG_baseline_. A significant correlation between tTLG_baseline_ and tRLU_dosage_ indicates that ^177^Lu-lilotomab satetraxetan targets FDG avid viable tumor cells as visualized by PET. **(c)** tRLU_dosage_ plotted against tRTV (day 4) shows that increasing tumor volume does not have a reducing effect on ^177^Lu-lilotomab satetraxetan uptake intensity. **(d)** ^177^Lu-lilotomab satetraxetan activity concentration tRLU ^dosage^ /volume plotted against SUV_mean_. Lacking correlation between tRLU ^dosage^/volume and baseline SUV_mean_ can be interpreted as glucose metabolism not necessarily correspond with CD37 expression. For all panels; blue dots represent data for individual patients and results from the Spearman-rank correlation tests are indicated in each panel.
