# Supplementary material for: FDG PET/CT and Dosimetric Studies of 177Lu-Lilotomab Satetraxetan in a First-in-Human Trial for Relapsed Indolent non-Hodgkin Lymphoma—Are We Hitting the Target?
Source: Mol Imaging Biol. 2022 Apr 29;24(5):807–17. doi: 10.1007/s11307-022-01731-3 (PMC9581842; doi:10.1007/s11307-022-01731-3)
Supplement: Supplementary file 2 — Supplementary file2 (DOCX 51 KB) [file 11307_2022_1731_MOESM2_ESM.docx]

| **Supplementary Table 1.** Detailed patient characteristics, treatment, PET and SPECT parameters, total tumor absorbed doses and clinical response. | 29 | M | 38 | 88/  177 | 2.04 | 5 | FL  I | 1728 | 20 | 122 | 536 | 4170 | 0 | 0 | 2 | 3 | 340 | 56.6 | 265 | 32.0 | 380 | CR | ^a^Patient did not have CeCT and FDG PET at 3 and 6 months. |
| --- | --- | --- | --- | --- | --- | --- | --- | --- | --- | --- | --- | --- | --- | --- | --- | --- | --- | --- | --- | --- | --- | --- | --- |
|  | 27 | M | 73 | 85/  180 | 2,05 | 4 | FL  IIIA | 1769 | 20 | 205 | 403 | 3680 | - | - | - | - | 428 | 45.7 | 260 | 21.3 | 240 | NE^a^ |  |
|  | 25 | M | 72 | 110/  176 | 2.24 | 4 | FL  I | 2189 | 20 | 224 | 131 | 743 | 8 | 92 | 39 | 333 | 249 | 22.1 | 208 | 12.8 | 210 | PR |  |
|  | 24 | F | 75 | 65/  163 | 1,70 | 4 | FL  I | 1290 | 20 | 174 | 219 | 1034 | 178 | 947 | 215 | 1158 | 285 | 7.1 | 263 | 3.9 | 60 | SD |  |
|  | 23 | M | 72 | 72/  180 | 1,91 | 4 | MCL | 1434 | 20 | 195 | 64 | 275 | 39 | 161 | 76 | 258 | 182 | 6.5 | 123 | 3.0 | 80 | SD |  |
|  | 22 | M | 74 | 56/  168 | 1,67 | 4 | FL  II | 1147 | 20 | 172 | 215 | 1952 | 107 | 1091 | - | - | 364 | 21.9 | 314 | 12.0 | 140 | SD |  |
|  | 21 | M | 62 | 93/  177 | 2,07 | 4 | FL  II | 1769 | 20 | 212 | 153 | 901 | 0 | 0 | 0 | 0 | 193 | 23.4 | 133 | 11.6 | 270 | CR |  |
|  | 20 | F | 75 | 58/  155 | 1,54 | 4 | FL  II | 1173 | 20 | 157 | 44 | 356 | 26 | 299 | 78 | 834 | 114 | 6.4 | 87 | 3.4 | 130 | PD |  |
|  | 19 | M | 70 | 85/  173 | 1,99 | 4 | FL  I | 1286 | 15 | 208 | 585 | 3884 | 466 | 3192 | 394 | 2537 | 531 | 45.4 | 397 | 21.4 | 190 | SD |  |
|  | 9 | M | 65 | 111/  187 | 2,35 | 1 | FL  II | 1696 | 15 | 48 | 63 | 975 | - | - | - | - | 113 | 5.7 | 41 | 1.7 | 130 | PD |  |
|  | 8 | M | 78 | 78/  174 | 1,9 | 1 | FL  I | 1220 | 15 | 48 | 175 | 515 | 0 | 0 | 0 | 0 | 219 | 7.0 | 202 | 3.2 | 70 | CR |  |
|  | 7 | M | 42 | 75/  171 | 1,86 | 1 | FL II | 1505 | 20 | 48 | 110 | 475 | 0 | 0 | 0 | 0 | 39 | 7.3 | 27 | 3.6 | 420 | CR |  |
|  | 5 | M | 70 | 97/  179 | 2,17 | 1 | FL  II | 1982 | 20 | 48 | 289 | 1540 | 14 | 37 | 8 | 27 | 205 | 9.8 | 223 | 6.3 | 120 | CR |  |
|  | 3 | M | 51 | 73/  171 | 1,85 | 1 | FL  II | 746 | 10 | 48 | 97 | 434 | 54 | 202 | 91 | 368 | 204 | 6.6 | 125 | 2.9 | 70 | SD |  |
|  | 2 | M | 69 | 103/  180 | 2,23 | 1 | FL  II | 1036 | 10 | 48 | 94 | 469 | 4 | 16 | 10 | 36 | 67 | 1.1 | 85 | 0.7 | 40 | PR |  |
|  | **Subject** | **Gender** | **Age** | **Patient weight (kg) / height (cm)** | **Patient BSA (m^2^)** | **Arm** | **Histology (grade)** | **Injected activity**  **(MBq)** | **Dosage level (MBq/kg)** | **Pre-dose**  **(mg)** | **tMTV_baseline_ (cm3)** | **tTLG_baseline_ (g)** | **tMTV_3months_(cm3)** | **tTLG_3months_ (g)** | **tMTV_6months_ (cm3)** | **tTLG_6months_ (g)** | **tRTV_day4_ (cm3)** | **tRLU_day4_ (MBq)** | **tRTV_day7_ (cm3)** | **RLU_day7_ (MBq)** | **tTAD (cGy)** | **Response** |  |
